# Stroke Risk After On-Pump Minimally Invasive Multivessel Coronary Artery Bypass Grafting With Right Axillary Artery Cannulation and Preoperative Computed Tomography Angiography

**DOI:** 10.1093/icvts/ivag257

**Published:** 2026-09-15

**Authors:** Christian Sellin, Hilmar Dörge, Marius Grossmann, Alexandru Durutya, Ardawan Rastan, Niki Tabesh, Volodymyr Demianenko

**Affiliations:** Department of Cardiothoracic Surgery, Heart-Thorax Center, Klinikum Fulda, University Medicine Marburg, Campus Fulda, 36043 Fulda, Germany; Department of Cardiothoracic Surgery, Heart-Thorax Center, Klinikum Fulda, University Medicine Marburg, Campus Fulda, 36043 Fulda, Germany; Department of Cardiothoracic Surgery, Heart-Thorax Center, Klinikum Fulda, University Medicine Marburg, Campus Fulda, 36043 Fulda, Germany; Department of Radiology, Klinikum Fulda, University Medicine Marburg, Campus Fulda, 36043 Fulda, Germany; Department of Heart and Cardiovascular Surgery, Philipps-University Marburg, 35043 Marburg, Germany; Department of Cardiothoracic Surgery, Heart-Thorax Center, Klinikum Fulda, University Medicine Marburg, Campus Fulda, 36043 Fulda, Germany; Department of Cardiothoracic Surgery, Heart-Thorax Center, Klinikum Fulda, University Medicine Marburg, Campus Fulda, 36043 Fulda, Germany

**Keywords:** minimally invasive cardiac surgery, coronary artery bypass grafting, cardiopulmonary bypass, axillary artery cannulation, stroke

## Abstract

**Objectives:**

Stroke remains a major concern in on-pump minimally invasive coronary artery bypass grafting (CABG), particularly when peripheral arterial cannulation and aortic manipulation are required in patients with systemic atherosclerosis. This study evaluated stroke outcomes after total coronary revascularization via left anterior thoracotomy (TCRAT) with preoperative computed tomography angiography (CTA) and right axillary artery (RAA) cannulation.

**Methods:**

A total of 800 consecutive patients undergoing isolated non-emergent multivessel minimally invasive CABG were analysed. All procedures were performed on cardiopulmonary bypass with RAA cannulation, transthoracic aortic cross-clamping, cardioplegic arrest, cardiac slinging manoeuvres, and side-bite clamping for proximal anastomoses. Of these, 85.4% were male; median age was 67.0 years (interquartile range 13.0), and median EuroSCORE II was 2.0 (interquartile range 2.1). All patients underwent preoperative CTA. Stroke events were analysed by timing, distribution, and presumed mechanism.

**Results:**

The overall in-hospital rate of major adverse cardiac and cerebrovascular events was 1.9%. In-hospital mortality was 1.0%, myocardial infarction 0.4%, repeat revascularization 0.6%, and stroke 0.6%. All strokes were ischaemic. Two strokes (0.25%) occurred within 72 hours and were considered procedure-related, whereas 3 (0.37%) occurred after 72 hours and were associated with postoperative atrial fibrillation or advanced intracranial atherosclerosis.

**Conclusions:**

In patients undergoing minimally invasive on-pump multivessel CABG after preoperative CTA confirmed suitable anatomy for transthoracic clamping and planned aortic manipulation, a standardized TCRAT strategy with RAA cannulation was associated with a low rate of clinically apparent in-hospital stroke. These findings support the feasibility and clinical rationale of this approach in routine practice, while comparative studies are required to define its independent effect on stroke prevention.

## Graphical abstract

**Figure:**
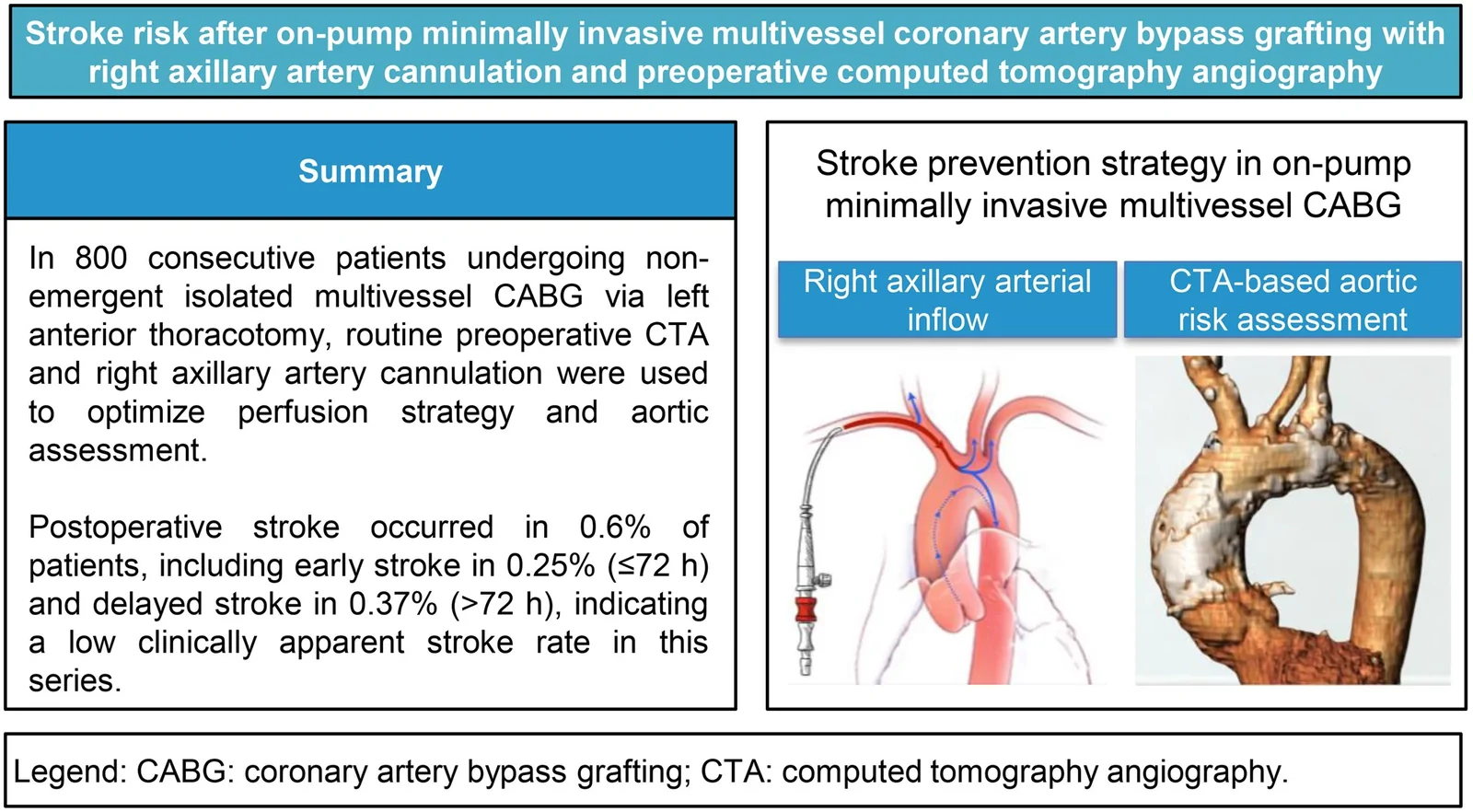

## INTRODUCTION

Stroke remains a devastating complication of coronary artery bypass grafting (CABG) and is associated with increased in-hospital mortality, prolonged recovery, and healthcare costs.^1^ Despite advances in perioperative care, it occurs in 0.8%-5.2% of cardiac surgery patients,^2^ with rates of approximately 1.2%-2.7% in isolated CABG.^3^^,^^4^ The aetiology is multifactorial and involves aortic manipulation during surgery, cerebral atheroembolism, and hypoperfusion. Intraoperative strokes, accounting for 40% of cases, are attributed to aortic manipulation, embolization from atherosclerotic plaques or hypoperfusion due to intracranial atherosclerotic disease (ICAD).^5^^,^^6^ Postoperative strokes occur within days after surgery and are linked to new-onset atrial fibrillation or pre-existing cerebrovascular disease. The distinction between these types is essential because they differ in mechanisms, prevention strategies, and impact on outcomes and long-term survival.^6^^,^^7^

Although on-pump CABG provides durable benefits in terms of myocardial infarction and cardiac survival over percutaneous coronary intervention (PCI) in multivessel coronary disease, its neurological risk remains a major limitation.^8–10^ In conventional CABG, the ascending aorta is the standard cannulation site; however, in minimally invasive, sternum-sparing procedures, peripheral cannulation is frequently required. Although widely used, femoral access may increase retrograde embolization and vascular injury, particularly in patients with peripheral vascular disease.^11^

The right axillary artery (RAA) offers an alternative cannulation site, providing antegrade cerebral perfusion and potentially reducing right-sided embolic risk by retrograde flow in the brachiocephalic trunk during cardiopulmonary bypass (CPB). Its safety and efficacy have been documented in acute type A aortic dissection^12^ and minimally invasive CABG, with excellent early and midterm outcomes and few access-related complications.^13^

In the setting of total coronary revascularization via left anterior thoracotomy (TCRAT), right axillary cannulation can be combined with preoperative computed tomography angiography (CTA) of the ascending aorta, facilitating safe cross-clamp placement and reliable antegrade cardioplegia delivery.^14^^,^^15^

The variability in stroke incidence and mechanisms underscores the need for procedure-specific data. Total coronary revascularization via left anterior thoracotomy combines complete coronary revascularization with the benefits of a minimally invasive approach, including reduced surgical trauma, shorter recovery, and improved patient satisfaction. However, the role of RAA cannulation in reducing perioperative stroke during TCRAT remains unclear. This study aimed to assess in-hospital stroke in patients undergoing TCRAT with RAA cannulation and preoperative CTA-based assessment.

## METHODS

This retrospective single-centre study was approved by the local ethics committee (Ethik-Kommission bei der Landesärztekammer Hessen; file number: 2021-2621-evBO; approval date: 28 December 2021) and conducted in accordance with the Declaration of Helsinki and the WMA Declaration of Taipei. The collection, storage, and use of routinely documented clinical data were covered by the ethics approval and subject to institutional data-protection governance and ethical oversight. Data were anonymized. Written informed consent for anonymized data analysis and publication was obtained from all patients. No biological material was collected or stored, and no biobank was used.

### Patient selection and procedural aspects

Between November 2019 and August 2025, isolated non-emergent CABG using the TCRAT technique was performed in 800 consecutive patients with multivessel coronary artery disease. Total coronary revascularization via left anterior thoracotomy, performed on CPB using peripheral arterial inflow via the RAA and cardioplegic arrest with transthoracic aortic cross-clamping, was the default strategy for multivessel coronary revascularization at our institution.

Patients requiring emergency surgery (same-day catheterization and operation) or redo procedures were excluded. Baseline characteristics are presented in **Table 1**.

**Table 1.** Baseline Demographic and Clinical Characteristics

Variable	Value (*n* = 800)
Age, years	67.0 (13.0)
Male sex	683 (85.4%)
Body mass index, kg/m²	27.4 (5.9)
Hypertension	793 (99.1%)
Diabetes mellitus	270 (33.8%)
Peripheral or cerebrovascular disease	338 (42.3%)
Prior stroke	30 (3.8%)
EuroSCORE II, %	2.0 (2.1)
Left ventricular ejection fraction, %	55.0 (10.0)
Two-vessel disease	173 (21.6%)
Three-vessel disease	627 (78.4%)
Left main stenosis >50%	273 (34.1%)
Prior NSTEMI	330 (41.3%)
Prior PCI	230 (28.8%)

Data are presented as median (interquartile range) or *n* (%).

Abbreviations: BMI, body mass index; LVEF, left ventricular ejection fraction; NSTEMI, non-ST-elevation myocardial infarction; PCI, percutaneous coronary intervention.

Preoperative CTA was performed before the final decision to proceed with TCRAT. All patients were selected for surgical revascularization following a multidisciplinary heart team discussion,^16^ with treatment determined according to guideline recommendations. As part of this process, consensus was reached regarding the coronary vessels requiring grafting based on anatomical complexity, the functional significance of stenoses, and the overall clinical presentation.^17^

All patients underwent preoperative CTA according to our institutional imaging protocol. This examination supplemented standard preoperative diagnostics to evaluate the ascending aorta, aortic arch, and major supra-aortic branches. The supra-aortic vessels, including carotid bifurcations, were assessed for relevant atherosclerosis during preoperative cerebrovascular risk evaluation. Significant carotid artery stenosis was defined as ≥70% luminal narrowing. The CTA protocol was designed to identify atherosclerotic plaques, calcification, and anatomical variations that could influence the cannulation strategy, aortic manipulation, and cross-clamping. In patients with impaired renal function, contrast-enhanced CTA was considered individually based on the severity and stability of renal dysfunction.

Patients were unsuitable for TCRAT if CTA demonstrated severe circumferential calcification of the ascending aorta, a porcelain aorta, or complex or protruding atheromatous plaque at an intended cross-clamp or side-bite clamp site (**Figure 1**).

**Figure 1.**
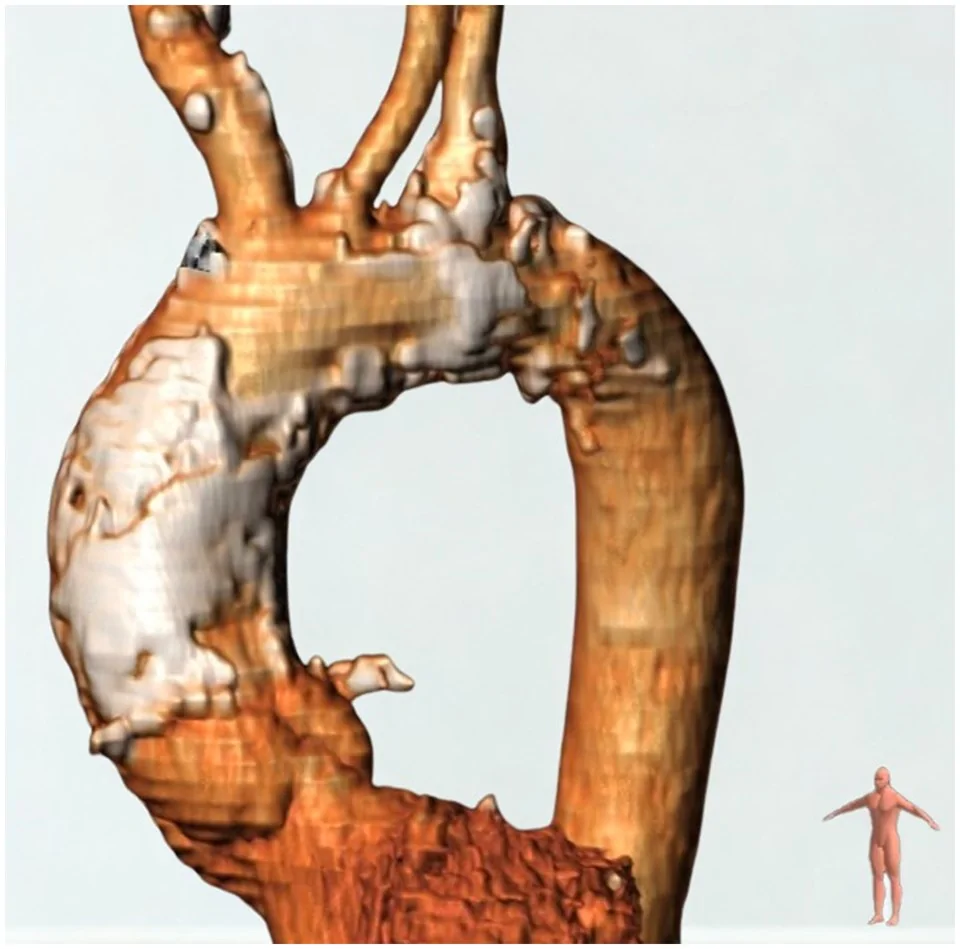
Contrast-Enhanced CTA of the Ascending Aorta Demonstrating Severe Circumferential Calcification

During the study period, 78 patients were excluded from TCRAT because CTA identified severe aortic disease and, after multidisciplinary heart team discussion, were redirected to alternative revascularization strategies such as conventional CABG with adapted aortic management, anaortic off-pump CABG, or PCI. Patients with limited focal calcification were not excluded from the minimally invasive strategy if preoperative CTA allowed clear identification of a safe site for aortic cross-clamp placement and other planned aortic manipulation.

Opioid-based multimodal anaesthesia was used with invasive monitoring and intraoperative TEE.

Technical aspects of TCRAT^18^^,^^19^ and direct RAA cannulation^13^ have been described previously. Right axillary artery cannulation was performed via limited surgical vessel exposure, placement of a purse-string suture, and guidewire insertion under direct vision.

Perioperative antiplatelet therapy followed contemporary guideline and consensus recommendations.^20^ Low-dose aspirin was continued preoperatively and restarted early postoperatively. Before elective CABG, ticagrelor, clopidogrel, and prasugrel were discontinued for at least 3, 5, and 7 days, respectively. Dual antiplatelet therapy was resumed postoperatively when clinically indicated and considered safe.

### Definitions and clinical end-points

The primary end-point was clinically apparent, imaging-confirmed in-hospital stroke. Secondary end-points included stroke timing (early or delayed), distribution, and presumed mechanism based on clinical context, neuroimaging, and preoperative vascular assessment. Other end-points included in-hospital mortality, myocardial infarction, repeat revascularization, and overall in-hospital major adverse cardiac and cerebrovascular events (MACCE).

Stroke was defined as a new permanent global or focal neurological deficit that could not be attributed to other neurological (eg, dementia) or medical causes (eg, metabolic abnormalities, hypoxia, or drugs).^7^ The diagnosis was established by neurological evaluation and confirmed by cerebral imaging. Neurological status was assessed during the early postoperative period, including within the first 24 hours after surgery. Any suspected focal or global neurological abnormality prompted neurological consultation and cerebral imaging, with further evaluation performed as clinically indicated. Confirmed stroke was managed in consultation with the neurological team based on current guidelines, imaging findings, and postoperative bleeding risk. Stroke was classified as early or delayed. Early stroke was defined as a new permanent neurological deficit occurring within 72 hours after surgery, whereas delayed stroke was defined as such an event occurring beyond the first 72 postoperative hours. This temporal distinction reflects different underlying pathophysiological mechanisms, such as intraoperative embolization in early stroke and postoperative atrial fibrillation or thromboembolic complications in delayed stroke.

Rhythm monitoring included continuous ECG during intensive or intermediate care and clinical assessment on the general ward. Postoperative atrial fibrillation was defined as new-onset atrial fibrillation documented by continuous ECG monitoring or 12-lead ECG during hospitalization.

Periprocedural myocardial infarction related to CABG was defined according to the Universal Definition of Myocardial Infarction (type 5 myocardial infarction)^21^ as a postoperative increase in cardiac troponin exceeding 10 times the 99th percentile upper reference limit within 48 hours after surgery, in combination with new electrocardiographic changes, imaging evidence of myocardial injury, or angiographic evidence of graft or native coronary artery occlusion.

### Statistical analysis

Clinical data were prospectively documented and retrospectively extracted from patient records. Continuous variables were assessed for normality and presented as medians (interquartile ranges) or means (standard deviations), as appropriate. Categorical variables were reported as *n* (%). Statistical analyses were performed using SPSS software (version 29.0.0.0; IBM SPSS Statistics, Armonk, NY, United States).

## RESULTS

Intraoperative and postoperative characteristics are summarized in **Table 2**. The left internal mammary artery was used in 98.6%, and multiple arterial revascularization was achieved in 60.4%. Among 11 patients without left internal mammary artery to left anterior descending coronary artery (LAD) grafting, 8 had no significant LAD disease, and 3 had intraoperative conduit constraints. Complete revascularization, defined by the preoperative surgical plan, was achieved in 758 patients (94.8%). Re-exploration for bleeding was required in 6.3%. Surgical revision was initially performed through the original left anterior thoracotomy; however, in 2 cases, satisfactory haemostasis could not be achieved through this access alone, requiring conversion to sternotomy. Preoperative CTA identified significant carotid artery stenosis in 28 patients (3.5%). No RAA cannulation-related vascular or upper-extremity neurological complications occurred. In-hospital MACCE was 1.9%, including 1.0% mortality, 0.4% myocardial infarction, 0.6% repeat revascularization (all by PCI; no redo CABG), and 0.6% stroke. During the same period, 183 additional patients underwent elective isolated CABG via median sternotomy for operational or resource-related reasons rather than CTA-defined anatomical unsuitability for TCRAT.

**Table 2.** Intraoperative and Perioperative Characteristics

Variable	Value (*n* = 800)
Aortic cross-clamp time, minutes	86.0 (42.0)
Cardiopulmonary bypass time, minutes	140.0 (52.0)
Operative time, minutes	298.0 (102.0)
Number of distal anastomoses	3.0 (1.0)
Left internal mammary artery used	789 (98.6%)
Radial artery used	470 (58.8%)
Saphenous vein used	525 (65.6%)
Multiple arterial revascularization	483 (60.4%)
Patients with proximal anastomoses on the ascending aorta	517 (64.6%)
Complete revascularization	758 (94.8%)
Postoperative atrial fibrillation	71 (8.9%)
Re-exploration for bleeding	50 (6.3%)
Myocardial infarction	3 (0.4%)
Repeat revascularization (PCI)	5 (0.6%)
Stroke	5 (0.6%)
Overall in-hospital MACCE	15 (1.9%)
In-hospital mortality	8 (1.0%)

Data are presented as median (interquartile range) or *n* (%).

Abbreviations: MACCE, major adverse cardiac and cerebrovascular events; PCI, percutaneous coronary intervention.

Among 800 patients undergoing isolated TCRAT, 5 postoperative ischaemic strokes (0.6%) occurred. Two strokes (0.25%) occurred early postoperatively (≤72 hours), whereas 3 (0.37%) were delayed (>72 hours). None of the patients with early or delayed stroke had significant preoperative carotid stenosis; stroke characteristics and carotid atherosclerotic findings are summarized in **Table S1**.

One early stroke involved the left posterior circulation. The second involved multifocal subcortical and cortical ischaemia within the right middle cerebral artery (MCA) and anterior cerebral artery (ACA) territories, with contralateral satellite lesions consistent with an embolic imaging pattern. In this patient, additional right jugular venous cannulation was required intraoperatively, and preoperative CTA demonstrated atherosclerotic plaque at the right carotid bifurcation (**Figure 2A and B**).

**Figure 2.**
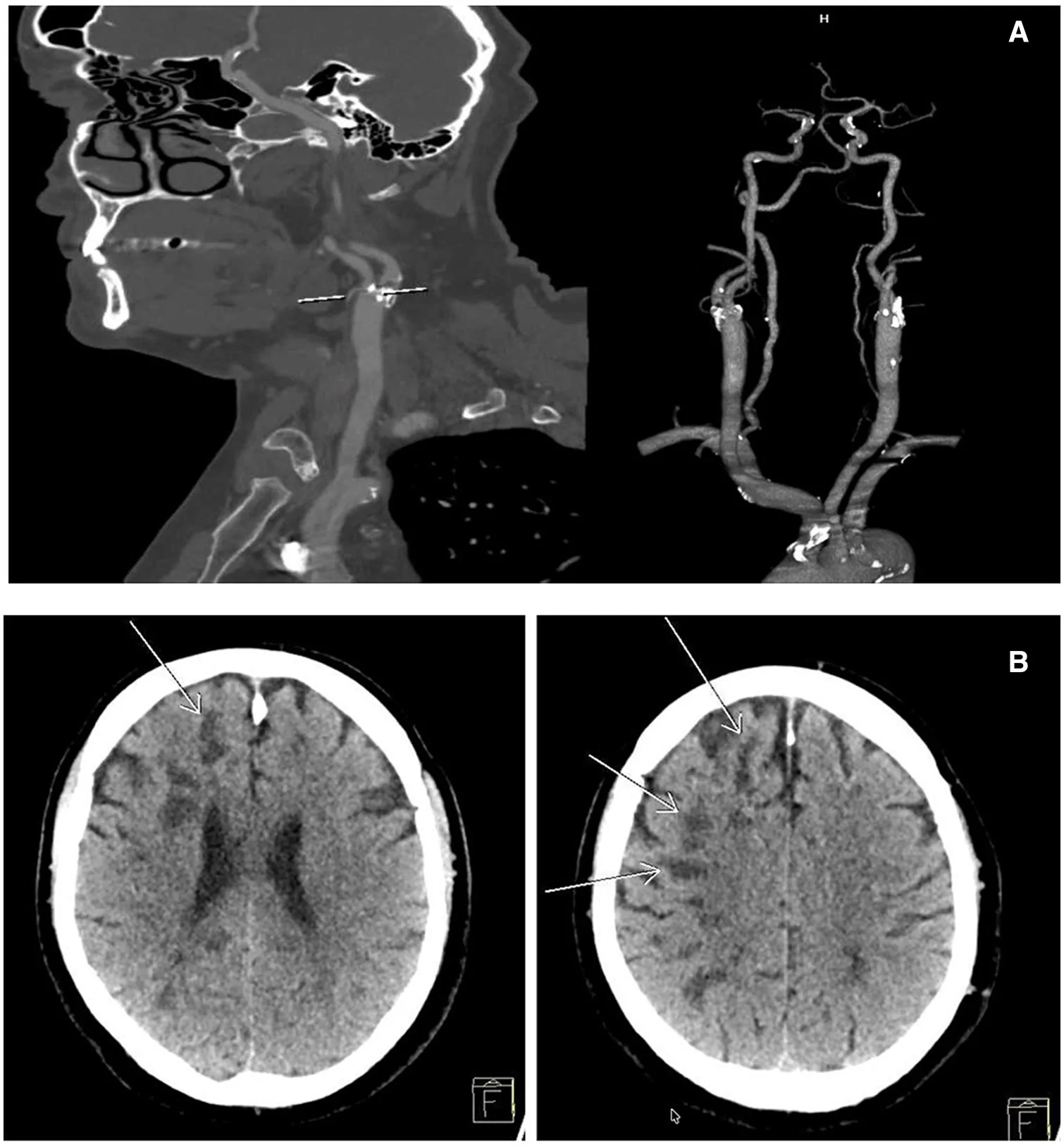
(A) Volume-rendered CTA of the aortic arch and supra-aortic vessels demonstrating marked atherosclerotic plaque at both carotid bifurcations. (B) Postoperative day 5 non-contrast CT showing multiple right MCA and ACA hypoattenuating foci consistent with subacute infarction

Delayed strokes had a different pathophysiological profile and were associated with underlying ICAD and vascular abnormalities. One patient presented with left vertebral artery occlusion and an additional MCA branch occlusion on a background of chronic infarct sequelae. Another patient exhibited multiple acute diffusion-restricted lesions confined to the left internal carotid artery territory in the presence of severe intracranial atherosclerosis and bilateral carotid bifurcation plaques. The third patient developed progressive occipital and occipitoparietal infarction with additional ischaemia of the right caudate nucleus in the setting of a hypoplastic basilar artery and bilateral embryonic posterior cerebral artery configuration.

## DISCUSSION

In this consecutive series of 800 patients undergoing isolated TCRAT, postoperative stroke occurred in 0.6%, including 2 early (0.25%) and 3 delayed (0.37%) events. This rate was lower than that reported after conventional CABG in major revascularization trials, although cross-trial comparisons should be interpreted cautiously.^8^^,^^10^^,^^22^ These findings suggest that targeted perfusion and imaging-based patient selection may contribute to a low perioperative stroke risk.

Off-pump CABG is often considered a strategy to reduce stroke by avoiding CPB. Among off-pump strategies, anaortic or “no-touch” CABG is frequently advocated as one of the most effective surgical approaches for stroke reduction because it avoids or markedly reduces manipulation of the ascending aorta. Stroke rates of 0.4%-0.7% have been reported with such techniques.^23–26^ However, no-touch aortic strategies are not universally applicable, particularly in minimally invasive multivessel revascularization.

Importantly, the neurological benefit of off-pump CABG has not been consistent across large randomized trials. The Randomized On/Off Bypass (ROOBY), German Off-Pump Coronary Artery Bypass Grafting in Elderly Patients (GOPCABE), and CABG Off or On Pump Revascularization Study (CORONARY) trials did not demonstrate a uniform reduction in stroke with off-pump surgery.^27–29^ Stroke rates ranged from 0.7% to 2.7% after on-pump CABG and from 1.0% to 2.2% after off-pump CABG, both exceeding the present rate. Similarly, a recent report from 2 high-volume centres (approximately 4000 patients) showed a stroke rate of about 2%, despite off-pump surgery in over 80% and automated devices for over 35% of proximal anastomoses.^30^

Off-pump minimally invasive coronary surgery may offer potential neurological advantages by avoiding CPB and, in anaortic configurations, manipulation of the ascending aorta. Minimally invasive direct coronary artery bypass (MIDCAB) is established for isolated LAD revascularization and may be combined with PCI as a hybrid strategy in selected patients, whereas multivessel minimally invasive cardiac surgery (MICS)-CABG extends this concept to more complex coronary disease. A recent systematic review suggested that MICS-CABG may be safe in well-selected patients with multivessel disease; however, interpretation of the available evidence is limited by heterogeneity in operative techniques, variable institutional experience, and the inclusion of large single-centre series from specific healthcare settings.^31^ Although off-pump MICS approaches represent important complementary strategies, their applicability depends on coronary anatomy, target accessibility, the feasibility of complete revascularization, and institutional expertise. They should therefore not be regarded as universally interchangeable alternatives to TCRAT.

The distinction between early and delayed stroke is clinically important because these subtypes differ in mechanisms and preventive implications.^6^^,^^7^ Early strokes are predominantly attributed to intraoperative embolization, often from the ascending aorta or retrograde perfusion during femoral cannulation, whereas delayed strokes are more commonly associated with postoperative atrial fibrillation, prothrombotic states, and ICAD.

The hemispheric distribution of cerebral lesions after cardiac surgery may provide insight into embolic routes.^32^ Early strokes reportedly predominate in the right hemisphere, whereas delayed events involved both hemispheres.^33^^,^^34^ In the present study, one early stroke involved the left posterior circulation, whereas the second early stroke showed a multifocal embolic pattern predominantly affecting the right MCA and ACA territories. Delayed strokes were more evenly distributed.

The early stroke rate was 0.25%. Right axillary artery cannulation may play an important role in this context. Right axillary artery cannulation preserves antegrade aortic and cerebral flow and may reduce the embolization of air or debris from the ascending aorta.^13^^,^^35–37^ An experimental cerebroprotective effect of RAA cannulation during CPB, mediated by altered flow patterns and retrograde flow within the brachiocephalic trunk, was previously demonstrated.^38^ This concept is supported by computational fluid dynamics simulations.^39^

Right axillary artery cannulation is widely used as an alternative peripheral perfusion strategy, particularly in high-risk settings such as acute type A aortic dissection, redo aortic surgery, and diffuse atherosclerosis of the ascending aorta.^40^ By directing flow towards the right common carotid artery and, indirectly, towards the left common carotid artery, this technique may reduce the likelihood of embolic events during manipulation of the ascending aorta.^37^^,^^38^

The proposed neuroprotective “one-way valve” mechanism is illustrated schematically in **Figure 3** and is particularly relevant in the context of aortic manipulation during TCRAT. In all patients, the ascending aorta was manipulated by transthoracic cross-clamping, and in 517 patients (64.6%), additional side-bite clamping was required for the construction of proximal anastomoses. These manoeuvres may be sources of particulate or air embolic material. During CPB, right axillary inflow directs blood towards the brachiocephalic trunk, generating retrograde flow towards the aortic arch. This flow direction may oppose the antegrade migration of embolic material released from the ascending aorta into the right common carotid artery. Meanwhile, cerebral perfusion is maintained through the carotid circulation, and systemic perfusion of the aortic arch and distal aorta is preserved. Thus, when combined with preoperative CTA-based identification of suitable sites for cross-clamping and side-bite clamping, RAA cannulation may provide a flow-directed haemodynamic barrier during aortic manipulation.

**Figure 3.**
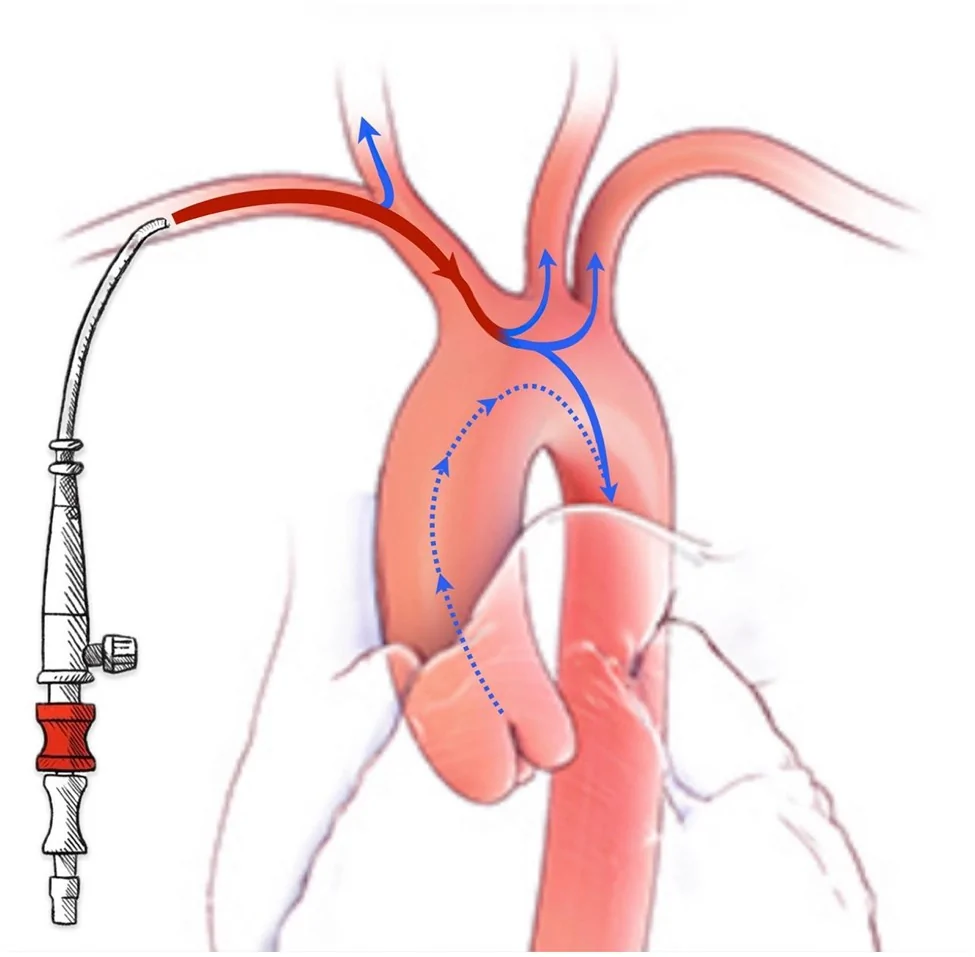
Schematic Illustration of the “One-Way Valve” Mechanism and Flow Direction. Blue indicates antegrade perfusion; red indicates retrograde perfusion. Retrograde flow within the brachiocephalic trunk may limit antegrade embolization towards the right carotid artery

Nevertheless, this haemodynamic concept should be interpreted in the context of individual vascular anatomy. Although right axillary arterial inflow maintains antegrade cerebral perfusion, the degree of retrograde flow within the brachiocephalic trunk may vary according to the aortic arch configuration, supra-aortic branching pattern, and presence of vascular disease. This further supports the role of preoperative CTA in assessing the ascending aorta, aortic arch, and supra-aortic vessels.

Preoperative CTA has received increasing attention in cardiac surgery.^14^^,^^15^ Reliable identification of extensive calcification or complex atheromatous plaque within the ascending aorta is particularly important in minimally invasive procedures. Previous studies show that preoperative CTA improves detection of relevant aortic pathology, facilitates technical modification, and may reduce perioperative neurological events, even in CABG via sternotomy. Computed tomography angiography may influence the cannulation site, cross-clamp strategy, and overall operative approach.

Epiaortic ultrasonography enables real-time detection of non-calcified and mobile ascending aortic plaques with greater sensitivity than manual palpation and transoesophageal echocardiography. Compared with epiaortic ultrasonography, CTA allows preoperative assessment of the entire aorta and additional non-vascular structures, thereby facilitating procedural planning and patient selection. Despite the associated radiation exposure, CTA remains useful in this setting.

Given the non-comparative design, these findings should be interpreted with caution. Further comparative studies are required to clarify the specific contribution of cannulation strategy and preoperative CTA to stroke outcomes in minimally invasive multivessel CABG.

### Limitations

Several limitations should be acknowledged. First, this was a retrospective, single-centre, non-comparative study; therefore, no causal inference can be made regarding the independent effect of RAA cannulation or routine preoperative CTA on stroke prevention, and superiority over alternative surgical strategies cannot be established. Second, the cohort comprised patients considered suitable for TCRAT after CTA-based anatomical assessment, limiting generalizability, particularly to patients with severe aortic disease or unfavourable anatomy for transthoracic cross-clamping and aortic manipulation. Third, the analysis was limited to in-hospital outcomes and did not include standardized post-discharge neurological follow-up using validated functional scales; therefore, long-term neurological recovery and post-discharge neurological events could not be systematically assessed. Finally, neurological assessment was based on routine postoperative clinical monitoring, with neurological consultation and cerebral imaging performed when clinically indicated rather than on standardized neurological scoring or systematic postoperative brain imaging in all patients. Although stroke timing was based on clinical recognition within a standardized in-hospital pathway, rare early cerebral events with delayed manifestations could theoretically have been diagnosed beyond 72 hours. Accordingly, the reported stroke rate reflects clinically apparent, imaging-confirmed in-hospital stroke and may underestimate clinically silent cerebral infarction or subtle neurological deficits.

## CONCLUSION

In this retrospective single-centre series, a standardized TCRAT strategy combining routine preoperative CTA-based vascular assessment with RAA cannulation was associated with a low rate of clinically apparent in-hospital stroke in patients undergoing minimally invasive on-pump multivessel CABG. The results highlight the practical value of systematic vascular imaging and antegrade peripheral arterial inflow as part of routine procedural planning for minimally invasive multivessel coronary surgery. Given the non-comparative design, these findings should be interpreted as hypothesis-generating and require confirmation in comparative studies.

## Supplementary Material

ivag257_Supplementary_Data

## Data Availability

The data underlying this article will be shared upon reasonable request to the corresponding author.

## ABBREVIATIONS

ACA: anterior cerebral artery
CABG: coronary artery bypass grafting
CPB: cardiopulmonary bypass
CTA: computed tomography angiography
ICAD: intracranial atherosclerotic disease
MCA: middle cerebral artery
PCI: percutaneous coronary intervention
RAA: right axillary artery
TCRAT: total coronary revascularization via left anterior thoracotomy
