# Supplementary material for: Stroke Risk After On-Pump Minimally Invasive Multivessel Coronary Artery Bypass Grafting With Right Axillary Artery Cannulation and Preoperative Computed Tomography Angiography
Source: Interdiscip Cardiovasc Thorac Surg. 2026 Sep 15;41(11):ivag257. doi: 10.1093/icvts/ivag257 (PMC13730571; doi:10.1093/icvts/ivag257)
Supplement: Supplementary file 1 [file ivag257_supplementary_data.zip › Supplementary Table S1.docx]

**Supplementary Material**

**Supplementary Table S1. Clinical and imaging characteristics of patients with postoperative stroke.**

This table summarizes the clinical presentation, neuroimaging findings, and presumed mechanisms of postoperative stroke in patients undergoing total coronary revascularization via left anterior thoracotomy.

| **Case** | **Stroke Type** | **Clinical Presentation** | **Neuroimaging Findings** | **Interpretation** |
| --- | --- | --- | --- | --- |
| **1** | Early postoperative | Symptoms compatible with posterior circulation involvement. | Left posterior circulation infarct; chronic right MCA infarct; microangiopathy; no hemorrhage. | New posterior circulation infarct on background of prior right MCA stroke. |
| **2** | Early postoperative | Focal deficits suggestive of multifocal embolic ischemia. | Multiple ischemic lesions in right MCA/ACA territories; small contralateral lesions; no hemorrhage. | Perioperative embolic ischemia. |
| **3** | Delayed postoperative | Facial droop and reduced responsiveness. | Chronic MCA defects; right vertebral artery occlusion; right M3 branch occlusion; possible new left temporal lesion. | Delayed ischemic event with vertebral artery occlusion and MCA branch occlusion. |
| **4** | Delayed postoperative | Deficits consistent with left ICA-territory ischemia. | Multiple acute DWI lesions in left ICA territory; carotid plaques; moderate right ICA stenosis; intracranial arteriosclerosis. | ICAD-related multiple embolic lesions on background of generalized atherosclerosis. |
| **5** | Delayed postoperative | Visual deficits and mild motor slowing (posterior circulation + basal ganglia pattern). | Right occipital and caudate infarcts; progression on follow-up; no hemorrhage; hypoplastic basilar artery; embryonic PCAs; systemic atherosclerosis. | ICAD-related posterior circulation and basal ganglia ischemia, enhanced by congenital vascular variants. |

**Abbreviations:** ACA, anterior cerebral artery; BA, basilar artery; CTA, computed tomographic angiography; CT, computed tomography; DWI, diffusion-weighted imaging; ICA, internal carotid artery; ICAD, intracranial atherosclerotic disease; LVO, large-vessel occlusion; MCA, middle cerebral artery; PCA, posterior cerebral artery; VA, vertebral artery; M3 branch, insular (third-order) branch of the MCA.
